# Supplementary material for: Proteomics Analysis Reveals that Warburg Effect along with Modification in Lipid Metabolism Improves In Vitro Embryo Development under Low Oxygen
Source: Int J Mol Sci. 2020 Mar 14;21(6):1996. doi: 10.3390/ijms21061996 (PMC7139666; doi:10.3390/ijms21061996)
Supplement: Supplementary file 1 [file ijms-21-01996-s001.zip › Table S5.docx]

Table S5. Details of the primer pairs use for Sybr green based real time qPCR

| Gene | GenBank Accession number | Primer sequence | Product size (bp) | Efficiency |
| --- | --- | --- | --- | --- |
| RPS15 | XM_006050525.2 | PF:5'- ACAACGGCAAGACCTTCAAC -3' | 153 | 105.40 |
|  |  | PR:5'- CAGGTTACTTGAGGGGGATG -3' |  |  |
| GAPDH | XM_006065800.2 | PF:5'-CAGGTTGTCTCCTGCGACTT-3' | 83 | 99.13 |
|  |  | PR:5'-AAGTGGTCGTTGAGGGCAAT-3' |  |  |
| PFK-1 | XM_025284056.1 | PF:5'- GGGCCTGGTGTTAAGGAACG -3' | 108 | 107.71 |
|  |  | PR:5'- ACGTTCTTCCTGCTGTCGAAG -3' |  |  |
| LDHA | XM_006056061.2 | PF:5'- TTGACAGTGCTTATGAGGTGATC -3' | 90 | 104.46 |
|  |  | PR:5'- TCATTATACTTTCTGCCAAATCGG -3' |  |  |
| GLUT3 | XM_006069760.2 | PF:5'- AAGATGCGGGTGTCCAAGAG-3' | 132 | 102.83 |
|  |  | PR:5'- CTCCAAGGCCAATCAGGTGT -3' |  |  |
