# Supplementary material for: Proteomics Analysis Reveals that Warburg Effect along with Modification in Lipid Metabolism Improves In Vitro Embryo Development under Low Oxygen
Source: Int J Mol Sci. 2020 Mar 14;21(6):1996. doi: 10.3390/ijms21061996 (PMC7139666; doi:10.3390/ijms21061996)
Supplement: Supplementary file 1 [file ijms-21-01996-s001.zip › Table S4.docx]

**Supplementary Table 4: List of Differentially Expressed Proteins**

| **Accession No.** | **Description** | **Log2 FC or m-Value** | **P-value** |
| --- | --- | --- | --- |
| A0A140T897 | Serum albumin OS=Bos taurus OX=9913 GN=ALB PE=4 SV=1 | 0.738344 | 6.28E-07 |
| A7Z064 | 3-hydroxy-3-methylglutaryl-coenzyme A reductase OS=Bos taurus OX=9913 GN=HMGCR PE=2 SV=2 | 0.650129 | 6.47E-08 |
| Q17QI3 | Acetyl-CoA acetyltransferase 2 OS=Bos taurus OX=9913 GN=ACAT2 PE=1 SV=1 | 0.535091 | 8.44E-06 |
| F1MBW3 | Acyl-CoA synthetase long chain family member 4 OS=Bos taurus OX=9913 GN=ACSL4 PE=4 SV=1 | 0.481604 | 6.19E-05 |
| Q3SZR3 | Alpha-1-acid glycoprotein OS=Bos taurus OX=9913 GN=ORM1 PE=2 SV=1 | 0.442111 | 0.000236 |
| Q32PF2 | ATP-citrate synthase OS=Bos taurus OX=9913 GN=ACLY PE=2 SV=1 | 0.432558 | 0.000324 |
| Q3MHE4 | DNA mismatch repair protein Msh2 OS=Bos taurus OX=9913 GN=MSH2 PE=2 SV=1 | 0.403549 | 0.000851 |
| A2VDL2 | Solute carrier family 2 (Facilitated glucose transporter), member 3 OS=Bos taurus OX=9913 GN=SLC2A3 PE=2 SV=1 | 0.393519 | 0.001085 |
| Q3T0P6 | Phosphoglycerate kinase 1 OS=Bos taurus OX=9913 GN=PGK1 PE=2 SV=3 | 0.38803 | 0.001265 |
| P19858 | L-lactate dehydrogenase A chain OS=Bos taurus OX=9913 GN=LDHA PE=2 SV=2 | 0.373982 | 0.001912 |
| F1MB08 | Alpha-enolase OS=Bos taurus OX=9913 GN=ENO1 PE=3 SV=1 | 0.373961 | 0.001912 |
| A5D984 | Pyruvate kinase OS=Bos taurus OX=9913 GN=PKM PE=1 SV=1 | 0.364271 | 0.002175 |
| F6RJG0 | 3-hydroxy-3-methylglutaryl coenzyme A synthase OS=Bos taurus OX=9913 GN=HMGCS1 PE=1 SV=1 | 0.358806 | 0.002899 |
| G3N3V1 | Phosphoglycerate mutase OS=Bos taurus OX=9913 PE=3 SV=1 | 0.325334 | 0.00619 |
| Q3ZBK2 | Redox-regulatory protein FAM213A OS=Bos taurus OX=9913 GN=FAM213A PE=2 SV=1 | 0.325265 | 0.007053 |
| P10096 | Glyceraldehyde-3-phosphate dehydrogenase OS=Bos taurus OX=9913 GN=GAPDH PE=1 SV=4 | 0.320037 | 0.007053 |
| Q3ZBE9 | Sterol-4-alpha-carboxylate 3-dehydrogenase, decarboxylating OS=Bos taurus OX=9913 GN=NSDHL PE=2 SV=1 | 0.319829 | 0.00802 |
| Q1LZA3 | Asparagine synthetase [glutamine-hydrolyzing] OS=Bos taurus OX=9913 GN=ASNS PE=2 SV=3 | 0.310622 | 0.008988 |
| Q5E956 | Triosephosphate isomerase OS=Bos taurus OX=9913 GN=TPI1 PE=2 SV=3 | 0.296284 | 0.012705 |
| Q2NL29 | Inositol-3-phosphate synthase 1 OS=Bos taurus OX=9913 GN=ISYNA1 PE=2 SV=1 | 0.267114 | 0.024675 |
| Q58DA3 | Ly1 antibody reactive OS=Bos taurus OX=9913 GN=LYAR PE=2 SV=1 | 0.267076 | 0.024675 |
| P08814 | Parathymosin OS=Bos taurus OX=9913 GN=PTMS PE=1 SV=2 | 0.24267 | 0.041323 |
| Q3MHP5 | Developmentally-regulated GTP-binding protein 1 OS=Bos taurus OX=9913 GN=DRG1 PE=2 SV=1 | 0.242549 | 0.041323 |
| Q9GMB8 | Serine--tRNA ligase, cytoplasmic OS=Bos taurus OX=9913 GN=SARS PE=2 SV=3 | 0.238243 | 0.045382 |
| A0A140T856 | Coatomer subunit alpha OS=Bos taurus OX=9913 GN=COPA PE=4 SV=1 | 0.238168 | 0.045382 |
| F2Z4H6 | Tripartite motif containing 23 OS=Bos taurus OX=9913 GN=TRIM23 PE=4 SV=1 | -0.23425 | 0.04978 |
| F1MNN6 | Major vault protein OS=Bos taurus OX=9913 GN=MVP PE=4 SV=1 | -0.23436 | 0.04978 |
| G3X7A8 | Uncharacterized protein OS=Bos taurus OX=9913 GN=LOC404103 PE=4 SV=1 | -0.25837 | 0.030414 |
| P01096 | ATPase inhibitor, mitochondrial OS=Bos taurus OX=9913 GN=ATP5IF1 PE=1 SV=2 | -0.26797 | 0.024675 |
| Q3MHL8 | Small acidic protein OS=Bos taurus OX=9913 GN=SMAP PE=2 SV=1 | -0.28278 | 0.019898 |
| G3N0V2 | Keratin 1 OS=Bos taurus OX=9913 GN=KRT1 PE=1 SV=1 | -0.31197 | 0.008873 |
| G3MX90 | Serine/threonine-protein phosphatase OS=Bos taurus OX=9913 PE=3 SV=1 | -0.31213 | 0.008873 |
| Q28107 | Coagulation factor V OS=Bos taurus OX=9913 GN=F5 PE=1 SV=1 | -0.31668 | 0.007916 |
| A6QNZ7 | Keratin 10 (Epidermolytic hyperkeratosis; keratosis palmaris et plantaris) OS=Bos taurus OX=9913 GN=KRT10 PE=2 SV=1 | -0.32139 | 0.024773 |
| F2Z4E7 | Interleukin enhancer binding factor 2 OS=Bos taurus OX=9913 GN=ILF2 PE=4 SV=1 | -0.33174 | 0.005423 |
| F1N650 | Annexin OS=Bos taurus OX=9913 GN=ANXA1 PE=3 SV=1 | -0.3457 | 0.003716 |
| A0A140T8D2 | Integrin beta OS=Bos taurus OX=9913 GN=ITGB1 PE=1 SV=1 | -0.37101 | 0.001879 |
| P11019 | V-type proton ATPase subunit E 1 OS=Bos taurus OX=9913 GN=ATP6V1E1 PE=2 SV=1 | -0.39053 | 0.001065 |
| F1MXZ0 | N-acetylglucosamine-6-sulfatase OS=Bos taurus OX=9913 GN=GNS PE=3 SV=1 | -0.42041 | 0.000433 |
| G3MZ71 | Keratin 2 OS=Bos taurus OX=9913 GN=KRT2 PE=1 SV=1 | -0.47919 | 7.63E-05 |
| Q0IIM3 | Heat shock protein 105 kDa OS=Bos taurus OX=9913 GN=HSPH1 PE=2 SV=1 | -0.49348 | 3.59E-05 |
| F1MUY2 | Uncharacterized protein OS=Bos taurus OX=9913 GN=KRT6B PE=1 SV=1 | -0.53284 | 9.81E-06 |
| P80724 | Brain acid soluble protein 1 OS=Bos taurus OX=9913 GN=BASP1 PE=1 SV=3 | -0.59959 | 5.70E-07 |
